# A 3D Indoor Modelling Method Using 360° Panoramic Images and Its Application to CCTV Camera Placement Optimization

**DOI:** 10.3390/s26113431

**Published:** 2026-05-28

**Authors:** Anak Agung Surya Pradhana, Nobuo Funabiki, I Nyoman Darma Kotama, Kadek Suarjuna Batubulan, Putu Sugiartawan

**Affiliations:** Department of Information and Communication Systems, Okayama University, Okayama 700-8530, Japan; p44c722y@s.okayama-u.ac.jp (A.A.S.P.); p9363bg2@s.okayama-u.ac.jp (I.N.D.K.); pzc37um1@s.okayama-u.ac.jp (K.S.B.); p18z9yov@s.okayama-u.ac.jp (P.S.)

**Keywords:** CCTV placement optimization, 360° panoramic image, 3D modelling, 3D Gaussian splatting, VSLAM, inertial measurement unit (IMU), ray-casting visibility analysis, greedy optimization, digital twin

## Abstract

Nowadays, *closed-circuit television (CCTV)* cameras are deployed worldwide to monitor movements of humans and other objects to improve the efficiency and safety of societies. Therefore, their proper placement is crucial for achieving effective surveillance coverage. Additionally, their proper placement is significantly important for maximizing *visual coverage* while reducing *installation/management costs*. For this task, *digital twin* is a useful technology, since it can simulate coverage and *blind spots* while freely changing camera locations. To implement *digital twin*, *3D modelling* of a structure including a complex room is a key issue. In this paper, we propose a *3D indoor modelling method* using *360° panoramic images* and show its application to a *CCTV camera placement optimization*. This method constructs a structured 3D model of a target room from captured 360° panoramic images using a *3D Gaussian Splatting* reconstruction method based on a *visual simultaneous localization and mapping (VSLAM)* framework. The *Inertial Measurement Unit (IMU)* is used together to improve the camera position estimation accuracy. The model construction is anchored using a *GNSS/GPS reference* to establish global spatial coordinates. As an application of the generated *3D model*, optimal locations of a given number of CCTV cameras are determined by combining *ray-casting visibility analysis* and a *greedy optimization algorithm* in the virtual environment, maximizing *visual coverage* while minimizing *blind spots* and avoiding excessive overlap between camera views. For evaluations, we applied the proposed method to three rooms in Okayama University, Japan, and seven rooms in the Indonesian Institute of Business and Technology, Indonesia. After optimizing camera locations in the virtual environment, the cameras were actually installed in the rooms according to the recommended positions. The performance was evaluated using *visibility coverage*, *blind spot reduction*, and *Root Mean Squared Error (RMSE)* between the estimated and actual camera positions, where promising results were achieved.

## 1. Introduction

Nowadays, *(CCTV)* cameras are widely deployed in various environments to monitor human activities and objects to improve the efficiency and safety of our societies [1,2]. Besides outdoor locations, CCTVs are commonly installed in indoor environments such as offices, university buildings, and public facilities. By continuously capturing visual information, CCTV systems enable surveillance operators to observe activities, detect *abnormal situations*, and support *security management* [3,4,5]. However, the effectiveness of a CCTV surveillance system strongly depends on the *placement of cameras* within the monitored environment. Proper camera placement is therefore essential to maximize the *visual coverage* of the target area while minimizing *blind spots* and avoiding unnecessary installation costs [6,7].

To support effective camera placement planning, *digital twin* technology has recently attracted attention. A digital twin represents a *virtual model* of a real environment that enables simulations before actual deployment [8,9]. In a CCTV system, it can simulate camera *fields of view* and estimate coverage areas under different placement configurations, helping to determine camera locations that can maximize visibility coverage by minimizing blind spots [10,11].

To perform such a simulation, an accurate *three-dimensional (3D) model* of the target indoor room environment is required. Previous studies have explored indoor 3D modelling using technologies such as *laser scanners*, *RGB-D cameras*, and *VSLAM* [12,13]. Among them, vision-based methods using *panoramic images* have attracted attention because they can capture comprehensive environmental information with fewer images while enabling *camera trajectory estimation* and *spatial reconstruction* [14,15].

However, existing approaches have several limitations when applied to practical CCTV placement planning. First, some methods require specialized and expensive equipment, such as laser scanners, which increases deployment costs [16,17]. Second, other vision-based approaches suffer from reduced accuracy in *camera position estimation*, especially in indoor environments with complex structures [18,19]. Third, the integration of *indoor 3D modelling* with practical applications, such as *CCTV camera placement optimization,* remains insufficiently investigated [20,21,22].

In this paper, we propose a *3D indoor modelling method* using *360° panoramic images* and demonstrate its application to the CCTV camera placement optimization within a digital twin environment. The proposed method constructs a structured 3D model of a target room from captured panoramic images using a *3D Gaussian Splatting* reconstruction approach integrated with a *VSLAM-based pose estimation* framework, supported by *IMU* and *GNSS/GPS* data to improve spatial accuracy.

To address existing limitations, this paper introduces an *end-to-end indoor modelling pipeline* that integrates panoramic image acquisition, sensor-based localization, and 3D reconstruction to generate an accurate digital twin of indoor environments. In addition, a *3D model refinement strategy* is proposed, including *point cloud upscaling* and *ceiling removal,* to improve the reliability of visibility analysis for surveillance applications. Furthermore, a *hybrid camera placement optimization* method is developed by combining *ray-casting visibility simulation* with a *greedy algorithm* to maximize visual coverage while minimizing blind spots and reducing overlap between cameras. Finally, the effectiveness of the proposed framework is validated through *real-world CCTV deployment,* demonstrating high positional accuracy and reliable monitoring performance.

For the evaluation of the proposed method, we implemented the system and applied it to three rooms in Okayama University, Japan, and seven rooms in Indonesian Institute of Business and Technology, Indonesia. The optimized camera placement using the obtained 3D model was implemented in the real environments. Then, the performance of this camera placement was evaluated by measuring the *visibility coverage*, *blind spot reduction*, and the *Root Mean Squared Error (RMSE)* between the estimated and actual camera positions [23]. The experimental results demonstrate that the proposed method effectively assisted the CCTV camera placement optimization using the generated 3D indoor model.

Finally, this paper is structured as follows. Section 2 provides a review of related work to 3D indoor reconstruction and camera placement optimization. Section 3 describes the design of the proposed system pipeline. Section 4 explains its implementation. Section 5 presents experimental results for evaluations. Section 6 concludes the paper and discusses possible directions for future work.

## 2. Related Works

In this section, we introduce relevant works in literature.

### 2.1. CCTV Camera Placement Optimization

In [24], Manuel et al. explored the use of *panoramic images* to capture indoor environments and support *digital environment representation*. Panoramic imaging enables the acquisition of comprehensive visual information with fewer images compared to conventional ones. However, this approach primarily focuses on *environment visualization* and does not explicitly address practical applications such as *CCTV camera placement optimization*.

In [25], Zhengyuan et al. proposed a *Building Information Modeling (BIM)-based framework* for CCTV placement optimization. Although this approach is effective in *structured environments*, such models are not always readily available in practical scenarios. Therefore, an efficient method for constructing *indoor 3D models* and applying them to CCTV placement optimization is still required.

In [26], Li et al. proposed a method for optimizing the selection and placement of security cameras with the objective of minimizing the *overall system cost*. Their approach considers factors such as *camera types*, *coverage requirements*, and *installation constraints* to determine an efficient camera configuration. The results demonstrated that the proposed method can reduce installation and maintenance costs while maintaining adequate *surveillance coverage*. However, this approach assumes the availability of an accurate *spatial representation* of the environment and does not address the problem of *3D model construction*.

In addition to model-based optimization approaches, several simplified CCTV placement strategies have also been utilized in practical surveillance systems. Common heuristic approaches include corner-based placement and uniformly distributed camera placement, where cameras are installed based on geometric assumptions without requiring a detailed 3D representation of the environment. These approaches are computationally efficient and easy to deploy.

However, in general, they cannot adequately consider indoor occlusions caused by walls, furniture, and other objects, which may reduce the actual surveillance coverage in complex indoor environments. Therefore, our visibility-driven optimization framework based on a reconstructed *digital twin* remains important for achieving more reliable CCTV placement in practical indoor scenarios.

### 2.2. Indoor 3D Modeling for Digital Twin

In [27], Pan et al. investigated *digital communication mechanisms* using data analysis and intelligent algorithms for *multimedia information processing*. Their study demonstrated that statistical and algorithm-based approaches can effectively analyse complex information systems and support *decision-making processes*. However, the study does not specifically address *spatial modelling* or *indoor environment reconstruction*.

In [28], Urrutia et al. explored the use of *digital twin technologies* to support analysis and decision-making in *smart city* environments. A digital twin represents a virtual model of real-world infrastructure that enables *simulation and evaluation* of different scenarios prior to real-world implementation. Such virtual environments provide a useful platform for analysing *spatial structures* and supporting planning tasks. However, this work focuses on *large-scale urban environments* rather than indoor spaces.

In [29], Qin et al. investigated *geo-information technologies* and *spatial data analysis* to represent real-world environments and support planning processes. These approaches utilize various *spatial datasets* to construct digital representations of environments. However, these studies do not focus on efficient *indoor 3D model construction* using *360° panoramic images* nor on their application to CCTV camera placement optimization in *digital twin environments*.

### 2.3. Panoramic Image-Based Environment Modeling

In [30], Tejada et al. proposed a framework that combines *360° image processing* and *LiDAR point clouds* to detect objects and monitor *spatial changes* in underground mining environments. Their method integrates panoramic imaging and *machine learning techniques* to improve *object detection* in *GNSS-denied areas*. However, this study focuses on object detection and *environmental monitoring* rather than indoor 3D reconstruction for CCTV camera placement optimization.

In [31], Han et al. proposed a method to enhance *panoramic image segmentation* in indoor construction sites using the *Segment Anything Model (SAM)*. Their approach improves segmentation accuracy by *iteratively refining object labels*. However, this method focuses on image segmentation and requires high computational resources, whereas it does not address *geometric reconstruction* for *spatial analysis*.

In [32], Xu et al. proposed an *intrusion detection and tracking system* using a *UAV* equipped with a 360° panoramic camera. The system employs an improved *YOLOv5-based model* to detect and track targets from aerial images. However, this approach focuses on *aerial surveillance* and requires specialized hardware, whereas it is not designed for *indoor 3D environment modeling* or CCTV placement optimization.

## 3. System Architecture

In this section, we present the architecture of the proposed system for *indoor 3D modeling* and *CCTV camera placement optimization*.

### 3.1. Overview

**Figure 1:**
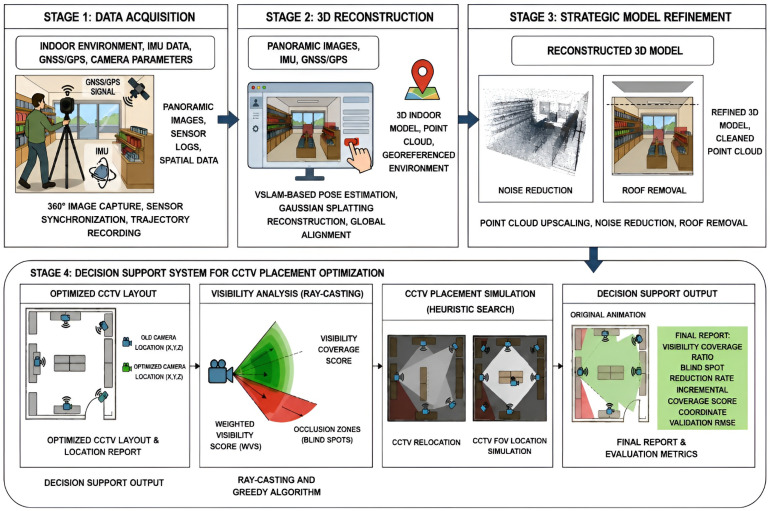
Workflow for *indoor 3D modelling* and *CCTV camera placement optimization.*

The proposed system follows a modular workflow, illustrated in Figure 1. This pipeline is organized with four stages: (1) *multimodal data acquisition*, (2) *spatial reconstruction*, (3) *geometric refinement*, and (4) *visibility-based optimization*. Unlike traditional approaches that treat modelling and camera placement as separate tasks, the proposed architecture tightly integrates these processes, ensuring that the generated 3D model is specifically tailored for *ray-casting-based visibility simulation*. By anchoring the virtual environment to global coordinates from the initial stage, the system enables a seamless transition from a digital twin to real-world deployment. The input, processing, and output for each stage are summarized in Table 1.

The four stages of our proposal together support data acquisition, 3D model reconstruction, its refinement, and CCTV placement optimization. This integrated design distinguishes the proposed system from existing indoor mapping approaches that focus mainly on visualization rather than surveillance analysis.

The overall processing pipeline consists of four sequential stages. First, synchronized panoramic images, *IMU* measurements, and *GNSS/GPS* metadata are collected during indoor data acquisition. Second, the captured images are processed using a *VSLAM* framework to estimate camera poses, where *IMU* information is integrated to improve the trajectory stability. Third, the estimated poses are utilized in the *3D Gaussian Splatting* reconstruction process to generate a dense point cloud representation of the indoor environment. Finally, the refined 3D model is evaluated through *ray-casting visibility simulation*, and a greedy optimization algorithm iteratively selects camera positions that maximize visibility coverage while minimizing blind spots and overlapping monitoring areas.

### 3.2. Data Acquisition and Sensor Fusion

This initial stage focuses on capturing the geometric structure of the target indoor environment. A *360° panoramic camera* is utilized to minimize the number of capture points while providing a *full spherical field of view.* To address the challenges in indoor positioning, where visual features may be repetitive or insufficient, a *sensor fusion approach* is employed.

In the proposed system, *IMU* measurements are utilized to provide motion and orientation information for improving the stability of camera trajectory estimation during the *VSLAM* process. This integration is particularly useful in indoor environments where visual features may be repetitive or partially occluded. Meanwhile, *GNSS/GPS* metadata is utilized as a global spatial reference to maintain coordinate consistency between the reconstructed virtual environment and the real-world environment. Although *GNSS/GPS* signals are limited in indoor environments, the recorded geospatial information still supports the alignment of the reconstructed model within a unified coordinate framework.

**Figure 2:**
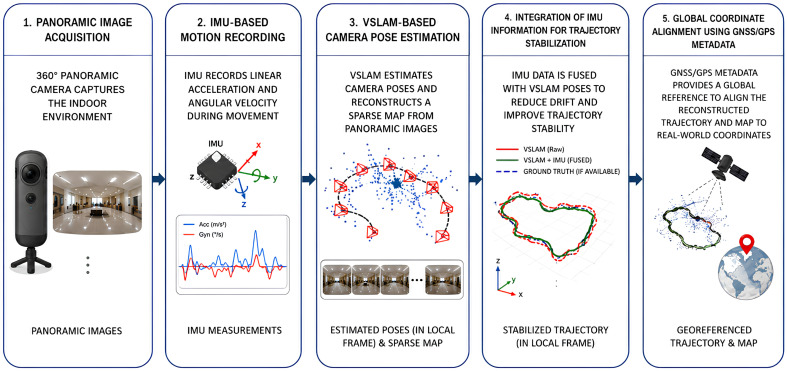
Sensor fusion workflow for VSLAM-based camera pose estimation, trajectory stabilization, and global coordinate alignment.

The sensor fusion process follows five sequential steps, as illustrated in Figure 2: (1) panoramic image acquisition, (2) IMU-based motion recording, (3) VSLAM-based camera pose estimation, (4) trajectory stabilization using IMU information, and (5) global coordinate alignment using GNSS/GPS metadata.

During the *VSLAM* process, feature points are extracted from consecutive panoramic images to estimate the relative camera motion. The *IMU* measurements are synchronized with the visual observations to provide motion priors for camera orientation and trajectory stabilization, especially in regions with limited visual features or temporary occlusions. After the local camera trajectory is estimated, the recorded *GNSS/GPS* metadata is utilized to align the reconstructed environment into a unified global coordinate system. The *IMU* mainly contributes to local trajectory refinement, while the *GNSS/GPS* information supports global spatial alignment between the virtual and real environments.

By synchronizing the panoramic images, *IMU* measurements, and *GNSS/GPS* metadata, the system records a robust camera trajectory that provides reliable spatial context for the subsequent reconstruction stage. This stage ensures that the collected data not only captures visual information but also provides reliable spatial references, which are essential for accurate reconstruction and consistent alignment with the real environment.

### 3.3. VSLAM-Based Indoor 3D Reconstruction

Based on the captured dataset, this stage reconstructs a coherent 3D representation of the indoor environment. A *visual simultaneous localization and mapping (VSLAM)* framework is employed to estimate camera poses accurately. To enhance both visual realism and geometric density, a *3D Gaussian Splatting* method is integrated into the reconstruction pipeline. This approach produces a continuous *point cloud* representation that effectively captures complex indoor structures, including occlusions caused by furniture and architectural elements in the room, which are essential for accurate visibility analysis. This stage plays a critical role in generating a structurally accurate digital twin that preserves both geometry and occlusions, which directly affect the reliability of subsequent visibility analysis.

### 3.4. Strategic Model Refinement

Before visibility analysis, this stage improves the reconstructed model through a *two-fold refinement process* for better quality and suitability to optimization. First, *point cloud upscaling* is applied to increase spatial density and fill in missing regions, preventing visibility rays from passing through sparse areas. Second, a *roof removal* procedure is performed. Since a CCTV system primarily focuses on monitoring activities at the *ground level*, removing ceiling structures prevents the optimization process from considering irrelevant surfaces. This refinement ensures that the analysis is concentrated on the *effective monitoring area* within the indoor environment. This stage is essential to transform the reconstructed model into a simulation-ready representation by eliminating irrelevant structures and improving geometric consistency for accurate visibility evaluation.

### 3.5. Greedy-Based CCTV Placement Optimization

The final stage determines the optimal camera configuration to maximize surveillance performance. Candidate camera positions are generated along the perimeter walls of the room. For each candidate, a *ray-casting algorithm* simulates the camera’s *line of sight* while accounting for occlusions caused by objects within the environment. Then, a *greedy optimization algorithm* is applied to iteratively select camera positions that maximize *incremental coverage,* defined as the additional visible area contributed by each new camera, while minimizing redundancy due to overlapping fields of view. The resulting configuration provides a *high-efficiency surveillance layout* that balances maximum coverage with minimal hardware deployment. This stage directly links the reconstructed digital twin with practical CCTV deployment by producing an optimized camera configuration that can be implemented in real-world environments.

In the visibility simulation, each virtual CCTV camera is modelled with a predefined viewing angle and visibility range that can approximate the practical field of view of a real surveillance camera. During the ray-casting process, occlusions caused by walls, furniture, and other indoor objects are considered to identify visible and non-visible regions within the environment.

In the optimization process, each candidate camera position is evaluated based on the number of newly visible points contributed to the monitoring area. At each iteration, the greedy algorithm selects the camera position with the highest incremental visibility gain. The process continues until the desired coverage threshold is achieved or no significant visibility improvement is obtained.

## 4. Implementation

In this section, we present the implementation of the proposed system for indoor 3D modelling and CCTV camera placement optimization using 360° panoramic images.

### 4.1. Panoramic Data Acquisition

In the data acquisition process, a *360° panoramic camera* mounted on a *camera stick* is used to capture comprehensive indoor spatial information. The use of a camera stick enables image capture from elevated viewpoints and narrow angles, allowing the system to record hidden or occluded areas that are difficult to observe from standard camera heights. This approach ensures that the entire indoor environment is captured without missing critical spatial details, which is essential for accurate *3D reconstruction* and reliable *CCTV placement analysis.*

**Figure 3:**
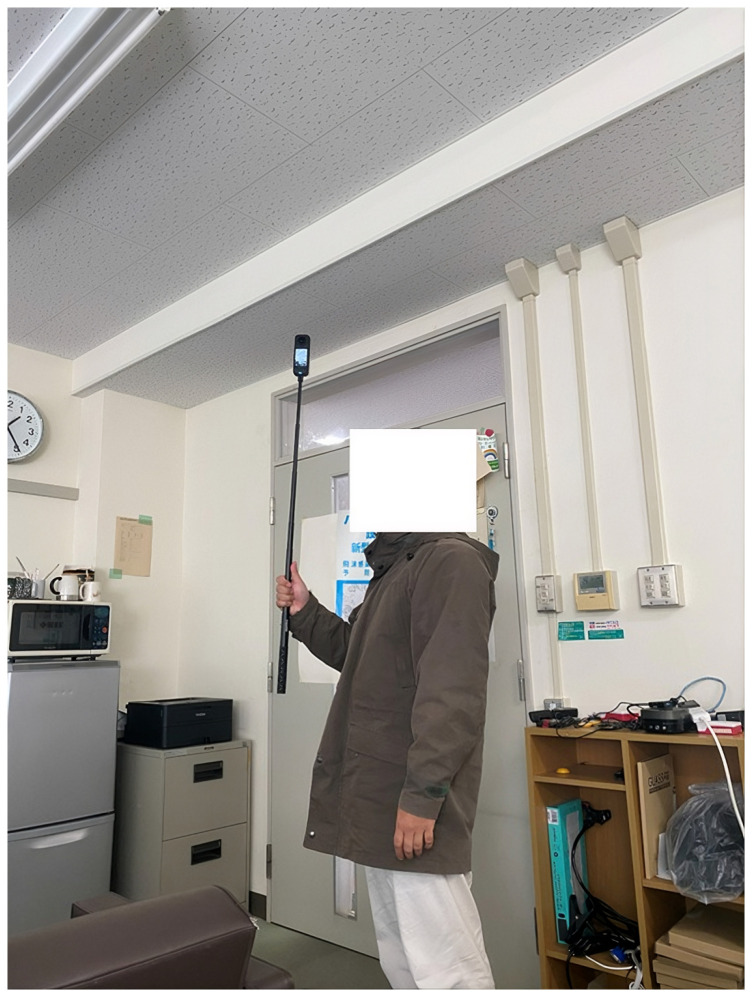
Data acquisition using 360° panoramic camera mounted on stick.

In addition, Figure 3 presents the camera device utilizes a built-in *Inertial Measurement Unit (IMU)* to record motion and orientation data during image acquisition. The *IMU* data improve the stability and accuracy of *camera pose estimation* in the subsequent reconstruction stage. Furthermore, a *GNSS/GPS reference* is incorporated to provide the global coordinate alignment, enabling the reconstructed indoor model to be anchored within a consistent *spatial reference frame*.

**Figure 4:**
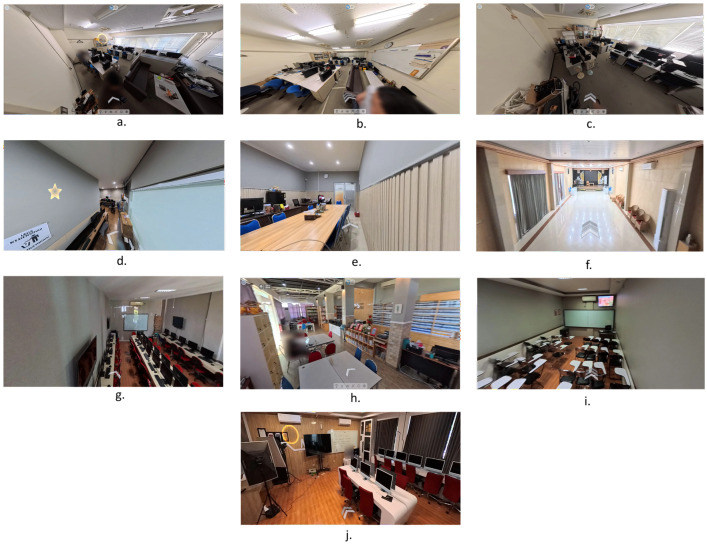
Samples of 360° panoramic datasets collected from various indoor environments with different layouts and characteristics: (**a**–**j**) represent different rooms used in this study.

Using a 360° panoramic camera with *IMU sensing* and *GNSS/GPS-based reference information,* the system captures detailed panoramic images of the entire room, including objects located in corners, behind furniture, and near walls. The completeness and stability of the collected data are crucial for generating a consistent indoor model that supports *digital twin construction* and subsequent CCTV placement optimization. Figure 4 presents sample panoramic images collected from multiple indoor environments with different layouts, sizes, and object distributions.

As summarized in Table 2, the dataset includes a variety of indoor environments with different sizes, layouts, and object densities. Rooms a-c belong to Okayama University, Japan, and rooms d-j belong to Indonesian Institute of Business and Technology, Indonesia. This diversity allows for evaluations of the proposed method under realistic and heterogeneous conditions, demonstrating its robustness and applicability to different indoor surveillance scenarios.

These rooms were selected as the target environments for CCTV installation experiments in this paper. The panoramic images were captured with a resolution of *5.7 K,* providing rich spatial information, including computers, desks, chairs, printers, and other indoor objects. This *high-resolution dataset,* combined with *IMU* and *GNSS/GPS*-supported pose estimation, is essential for constructing an accurate 3D indoor layout and enabling reliable *visibility simulation* in the CCTV placement optimization stage.

### 4.2. Data Processing and Modelling

The pipeline for transforming panoramic data into a 3D model and optimizing CCTV placement is presented here.

#### 4.2.1. 3D Environment Reconstruction Using 3D Gaussian Splatting

Following the data acquisition, the obtained panoramic images for each indoor room captured with a 360° panoramic camera are converted into a 3D representation of the room. In this paper, the 3D reconstruction is performed using a *3D Gaussian Splatting* method combined with a *VSLAM*-based pose estimation framework to generate a *dense point cloud* and a structured spatial model of the environment. The pose estimation is further enhanced by integrating *IMU* measurements and *GNSS/GPS*-based reference information acquired during the data acquisition stage, improving the stability and consistency of the reconstructed model.

Unlike conventional *mesh-based* or *voxel-based* reconstruction methods, the use of *3D Gaussian Splatting* enables a continuous and dense spatial representation that preserves *fine-grained geometric details.* This characteristic is particularly beneficial for visibility analysis, as it allows for more accurate *ray-casting simulation* in the subsequent CCTV placement optimization stage.

**Figure 5:**
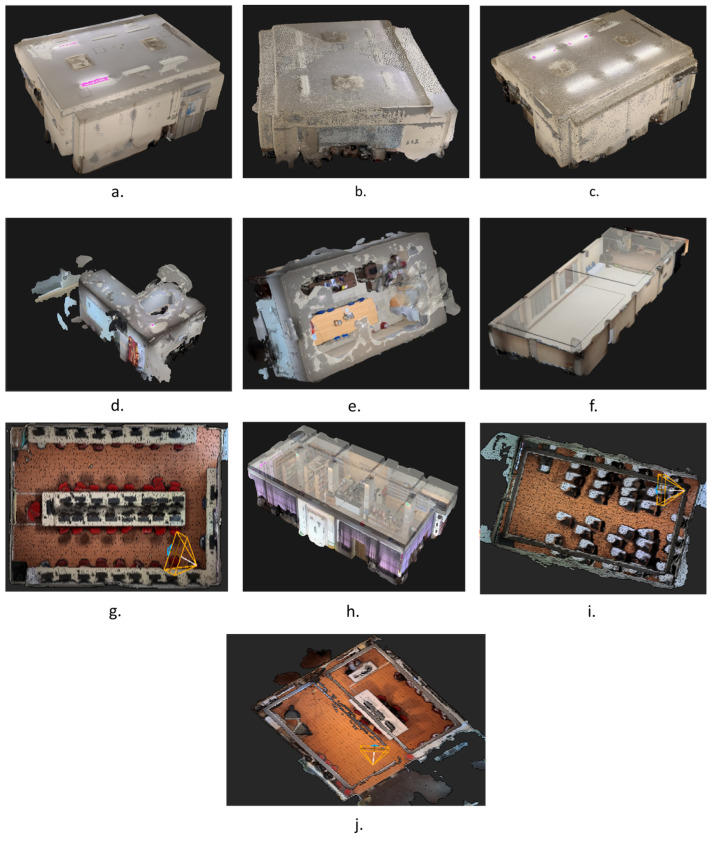
*3D reconstruction* and *point cloud generation* using *3D Gaussian Splatting.* (**a**–**j**) reconstructed indoor models generated from panoramic image datasets collected in different experimental environments.

As shown in Figure 5, the captured 360° panoramic images are transformed into a 3D *Gaussian Splatting* representation to generate detailed *point cloud data* of the indoor environment. This representation facilitates the identification of room structures and indoor objects, such as desks, computers, and other equipment. Moreover, the generated 3D model preserves both the *geometric layout* and the *spatial distribution* of the objects within the room, which is essential for reliable visibility analysis. Based on the reconstructed 3D spatial data, *candidate CCTV positions* can be evaluated in a virtual environment. The recommended camera locations are defined at appropriate coordinates within the point cloud, enabling accurate simulation of *camera fields of view* and supporting optimal visibility coverage analysis before real-world installation.

#### 4.2.2. 3D Model Refinement and Upscaling

After performing 3D *Gaussian Splatting*-based reconstruction, an *upscaling and refinement process* is applied to improve the quality of the generated 3D model. This process increases the density and consistency of the point cloud representation, resulting in a more detailed and accurate *spatial structure.*

This step is particularly important in the proposed system, as visibility analysis is highly sensitive to *surface continuity.* Sparse or incomplete point clouds may lead to incorrect *visibility estimation,* which can negatively affect the camera placement optimization results.

**Figure 6:**
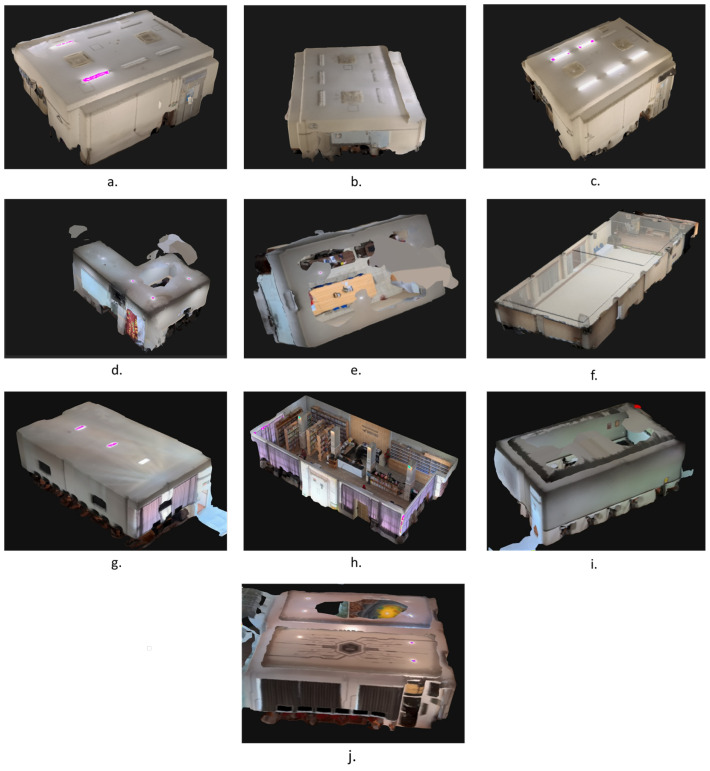
Improvement of the 3D model quality after the upscaling and refinement processes: (**a**–**j**) initial reconstructed models and refined models after upscaling and noise reduction.

As shown in Figure 6, the upscaling process enhances the visual quality of the reconstructed 3D environment by increasing *point density* and reducing missing or sparse regions in the point cloud. This refinement produces a more complete and consistent indoor representation, which is essential for accurate visibility simulation and CCTV placement optimization. By using a higher-quality 3D model, the system can evaluate candidate camera positions more precisely in the virtual environment.

#### 4.2.3. Ceiling Removal for Visibility Analysis

After improving the quality of the reconstructed 3D model, a *ceiling removal process* is applied before the CCTV placement simulation. This step is necessary because the optimization process needs to evaluate camera positions based on the *visible surface area* within the 3D environment. If the ceiling remains in the model, the optimization process may incorrectly select camera placements by focusing on the ceiling rather than the actual monitoring area.

This preprocessing step introduces a *task-oriented refinement strategy,* where non-relevant surfaces are explicitly removed to guide the optimization process toward meaningful monitoring regions. This differs from conventional reconstruction pipelines that preserve the complete geometry without considering *application-specific requirements.*

**Figure 7:**
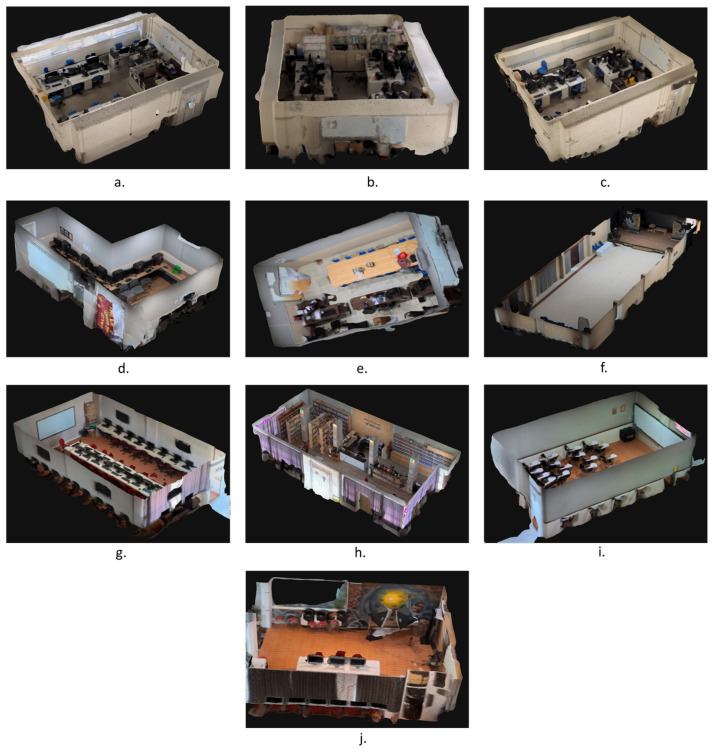
*Ceiling removal* from *3D model* for CCTV visibility analysis: (**a**–**j**) original reconstructed indoor models with ceiling structures and refined models after ceiling removal for visibility simulation.

As shown in Figure 7, the ceiling of the reconstructed room is removed to ensure that the visibility simulation focuses on the interior space where the surveillance is required. By eliminating the upper surface or ceiling, meaningful CCTV position candidates will be evaluated, enabling the proposal to prioritize camera placements on walls or corners where *effective monitoring* can be achieved. This step improves the reliability and accuracy of the subsequent visibility analysis and the camera placement optimization.

#### 4.2.4. Greedy-Based Camera Placement Using Ray-Casting Visibility Analysis

After preparing the refined 3D indoor model and removing the ceiling surface, the next step is to determine the optimal CCTV camera locations through visibility simulation. In this paper, a *ray-casting-based visibility analysis* combined with a *greedy optimization strategy* is employed to identify the optimal camera placement along the walls of the indoor environment. *Ray-casting* is used to simulate the field of the view of a virtual camera by projecting the rays from the candidate camera positions into the reconstructed 3D space. The rays are primarily directed toward lower-level surfaces, such as floors, desks, and equipment areas, which are regarded as the main monitoring targets. By computing the intersections between the projected rays and the point cloud surfaces, the *visible region* associated with each candidate camera position can be quantitatively estimated.

Based on the visibility estimation, a *greedy algorithm* is applied to iteratively select camera positions that maximize the *incremental visible surface coverage* while minimizing the redundancy caused by *overlapping fields of view*. At each iteration, our algorithm selects a camera location that contributes the maximum additional coverage to the previously covered area. This process continues until a desired coverage level is achieved or no significant improvement can be obtained.

**Figure 8:**
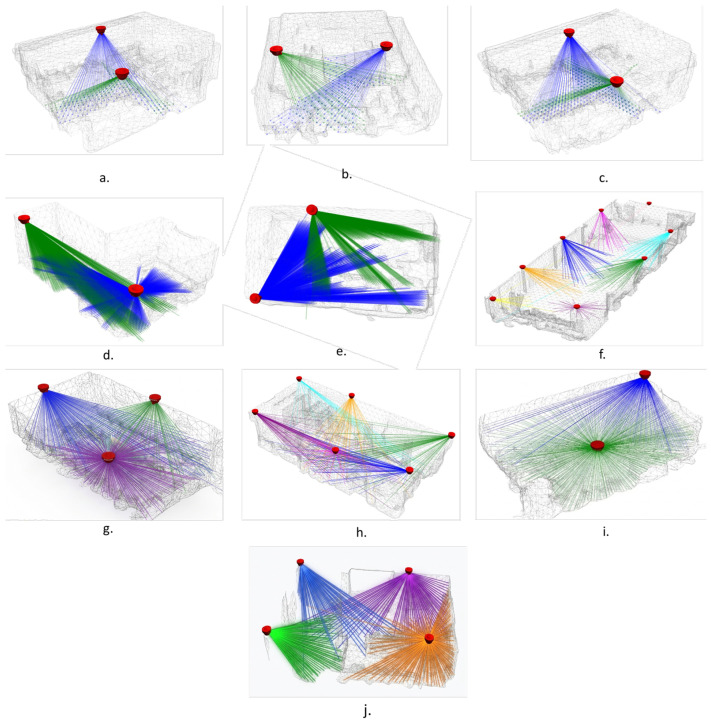
*Ray-casting-based* visibility simulation and *greedy camera placement optimization*: (**a**–**j**) visibility simulation results in different indoor environments and optimized CCTV camera placement configurations generated by the proposed method.

As shown in Figure 8, *candidate camera positions* are defined along walls of the reconstructed 3D model, and rays are projected toward interior surfaces to evaluate the visibility coverage. By integrating ray-casting analysis with a greedy optimization, the proposed method efficiently identifies camera configurations that maximize monitoring coverage while reducing *blind spots* and avoiding excessive overlap.

The integration of ray-casting visibility analysis with a greedy optimization strategy provides a *computationally efficient solution* to this camera placement problem. Compared to exhaustive search approaches, the proposed method significantly reduces computational complexity while achieving high coverage performance in practical indoor environments.

### 4.3. Implementation and Monitoring Result

Based on the optimal CCTV camera positions determined by the proposed ray-casting and greedy optimization method, the resulting configuration was implemented in real-world environments across 10 rooms in two universities. Cameras were installed at the optimal coordinates derived from the *3D digital twin model*, and the monitoring performance was subsequently validated using an actual CCTV system.

**Figure 9:**
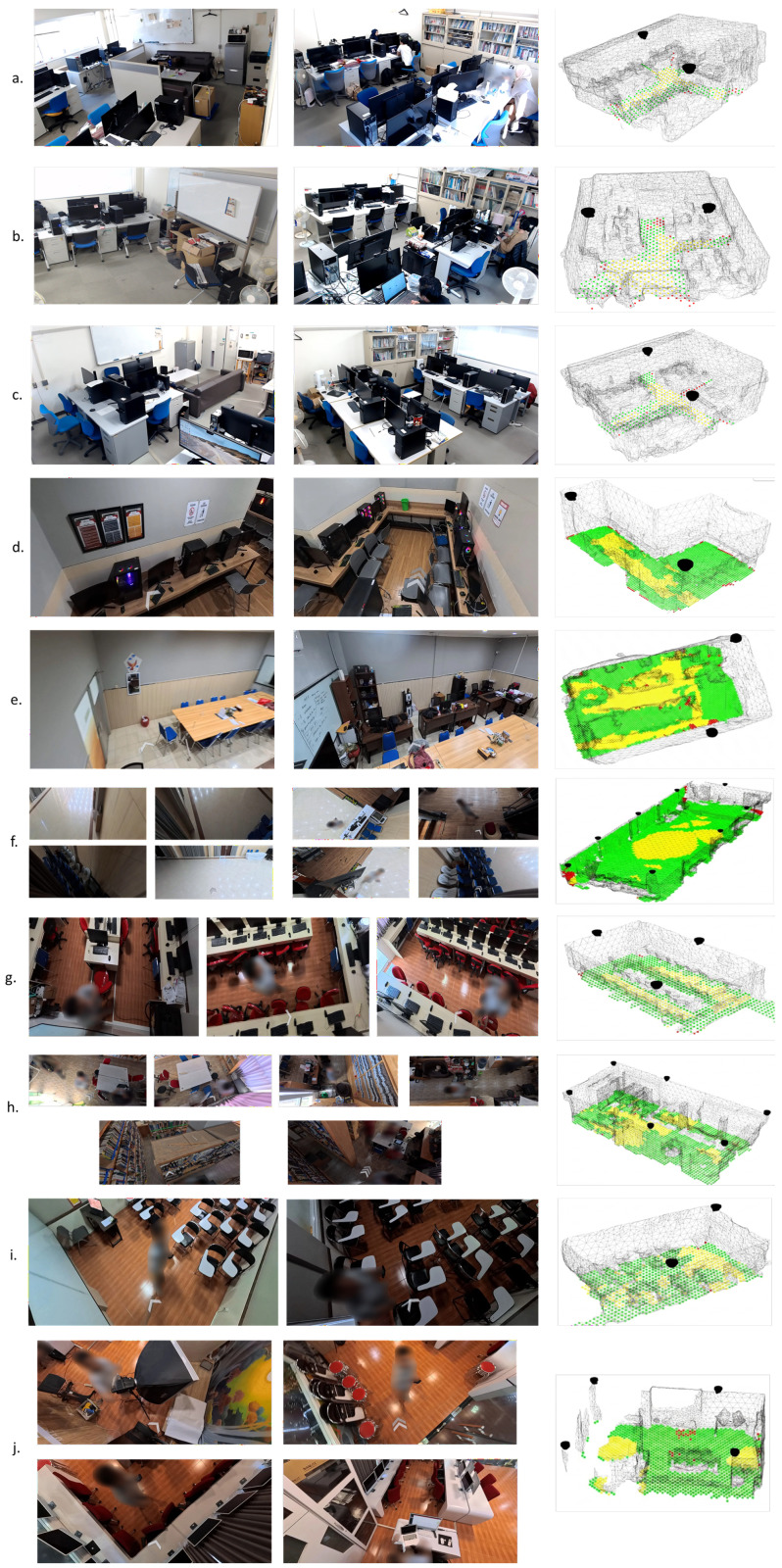
Real CCTV monitoring results after applying optimized camera placement in ten rooms: (**a**–**j**) monitoring views captured from the installed CCTV cameras in different experimental environments.

As shown in Figure 9, each room is presented with two types of visual results: (1) the real CCTV monitoring output with the reconstructed camera placement and (2) the visibility analysis result obtained using ray-casting. In the *visibility map*, coloured points indicate the level of coverage, where *green* represents *well-covered* areas, *yellow* indicates *moderately covered* areas, and *red* denotes *blind spots* or poorly observed regions.

The results demonstrate that the optimized camera placement achieves high coverage across the 10 rooms, as evidenced by the dominance of green regions. The remaining yellow and red areas are mainly located in occluded or structurally complex regions, such as behind furniture or near walls. Furthermore, the camera configuration effectively minimizes overlaps between camera fields of view while maintaining the comprehensive coverage of the monitored space. These findings indicate that the proposed method can generate a practical and directly deployable camera placement recommendation in a real-world environment. The consistency between the simulated visibility result and the actual CCTV monitoring output further confirms the effectiveness of the proposed *digital twin-based approach* for indoor surveillance applications.

## 5. Evaluation

In this section, we evaluate the proposed system on *visibility performance* and *position accuracy. Visibility coverage* and *blind spot reduction* assess surveillance effectiveness, while *position accuracy* is measured by comparing virtual and real camera positions.

### 5.1. Visibility Coverage Evaluation

First, *visibility coverage* and *blind spot reduction* by the proposal are evaluated. These metrics assess how well the optimized CCTV camera configuration monitors the indoor environment. *Visibility coverage* is defined as the proportion of the observable surface area within the indoor environment that is visible from the selected camera positions, while *blind spots* refer to regions that are not covered by any camera [10]. Based on these concepts, the *visibility coverage* and the *blind spot reduction* metric adapted from Baysal [33] are defined as follows:

(1) $$\mathrm{Coverage}=\frac{A_{\mathrm{visible}}}{A_{\mathrm{total}}}\times 100\%$$

(2) $$\mathrm{Blind}\;\mathrm{Spot}\;\mathrm{Reduction}=\frac{A_{\mathrm{initial}}-A_{\mathrm{uncovered}}}{A_{\mathrm{initial}}}\times 100\%$$

The computation of these metrics is conducted through a discrete *point cloud representation* of the reconstructed 3D environment. Let the indoor space be represented by a set of *M* surface points obtained from the *3D Gaussian Splatting* model after the refinement and *ceiling removal* processes. Each point is evaluated using *ray-casting* to determine its visibility from the selected camera positions.

A point is considered visible if at least one ray projected from any camera intersects the point without *occlusion.* Then, the visible area $A_{visible}$ is defined as the number of visible points. The total observable area $A_{total}$ corresponds to the total number of points in the environment.

For blind spot analysis, the initial area $A_{initial}$ is equal to $A_{total}$, representing the entire observable space before a camera placement. The uncovered area $A_{uncovered}$ represents the number of points that remain invisible after the camera placement optimization.

For example, in Room D207, if the total number of observable points is 10,000, among them, 9240 points are visible after optimization, and the coverage is calculated as:

(3) $$\mathrm{Coverage}=\frac{9240}{10,000}\times 100\%=92.4\%$$

Similarly, if the remaining uncovered points are 1490, the blind spot reduction is calculated as:

(4) $$\mathrm{Blind}\;\mathrm{Spot}\;\mathrm{Reduction}=\frac{10,000-1490}{10,000}\times 100\%=85.1\%$$

These computations are automatically performed during the *ray-casting visibility simulation,* allowing for the efficient and scalable evaluation of the coverage performance in a complex indoor environment.

As shown in Table 3, the proposed method achieves high visibility coverage across all experiment environments, with an average coverage of 93.5%. The *blind spot reduction rate* reaches an average of 87.0%, indicating that the optimization process effectively minimizes uncovered areas. These results demonstrate that this integration of *ray-casting visibility analysis* and *greedy optimization* can produce efficient CCTV camera configurations with comprehensive monitoring coverage in indoor environments.

### 5.2. Position Accuracy Evaluation

To evaluate the position accuracy of the proposed system, the camera locations obtained from the *virtual 3D model* are compared with the actual camera positions installed in the real indoor environments. This evaluation verifies whether the reconstructed *digital twin* can provide reliable spatial coordinates for practical CCTV deployments.

In this evaluation, the *RMSE* does not represent the manual installation error during the camera deployment. Instead, it measures the spatial consistency between the optimized camera positions generated in the virtual environment and the corresponding camera positions measured in the real indoor environment after implementation. Therefore, the *RMSE* reflects the alignment accuracy of the proposed *digital twin*-based reconstruction and optimization framework.

Experiments were conducted in three rooms in Okayama University, Japan, and seven rooms in the Indonesian Institute of Business and Technology, Indonesia. After generating the 3D model and performing the *camera placement optimization,* the system automatically determined the number of cameras required to achieve sufficient visibility coverage. The proposed method automatically determined the number of CCTV cameras required for each room based on the room size, layout complexity, and visibility coverage requirements. The proposed method recommended two CCTV cameras as the optimal configuration. The recommended camera positions obtained in the virtual environment were then implemented in the real rooms, and their coordinates were measured and compared with those estimated by the system. The *position precision* is evaluated using the *Root Mean Squared Error (RMSE)* according to the method of Kusuma [34], which is defined as:

(5) $$\mathrm{RMSE}=\sqrt{\frac{1}{N}\sum_{i=1}^{N}\left[{\left(x_{i}-x_{i}^{\prime }\right)}^{2}+{\left(y_{i}-y_{i}^{\prime }\right)}^{2}+{\left(z_{i}-z_{i}^{\prime }\right)}^{2}\right]}$$

where $\left(x_{i},y_{i},z_{i}\right)$ denotes a camera position in the virtual environment, $\left(x_{i}^{\prime },y_{i}^{\prime },z_{i}^{\prime }\right)$ denotes the actual measured position, and *N* is the number of cameras. The *RMSE* is computed as the average *Euclidean distance* between the estimated and actual positions [35]. For example, one camera in Room D207 had the estimated position (2.10,3.50,2.80) and the actual position (2.00,3.40,2.70), where the squared error is calculated as:

(6) $${\left(2.10-2.00\right)}^{2}+{\left(3.50-3.40\right)}^{2}+{\left(2.80-2.70\right)}^{2}=0.01+0.01+0.01=0.03$$

If two cameras are installed in one room, the *RMSE* is computed by averaging the squared errors of both cameras and taking the square root. For instance, if the total squared error for two cameras is 0.065, then:

(7) $$\mathrm{RMSE}=\sqrt{\frac{0.065}{2}}\approx 0.18\;m$$

The *RMSE* value is computed for any camera position in each room, and the results are summarized in Table 4. It compares the estimated camera positions from the virtual model with the actual positions in the real environment.

As shown in Table 4, the *RMSE* values are consistently low across all test environments, indicating that the camera positions generated by the *digital twin* are close to the actual installation positions. The average *RMSE* is 0.20 m, corresponding to an accuracy of approximately 95.7%. These results demonstrate that the proposed 3D reconstruction and optimization pipeline can generate a reliable *digital twin model* with sufficient accuracy for practical CCTV placement in real indoor environments.

### 5.3. Experimental Setup

The experiments were conducted in three rooms in Okayama University, Japan, and seven rooms in Indonesian Institute of Business and Technology, Indonesia. Each room contains typical office equipment such as desks, chairs, computers, and printers, representing a standard indoor working environment. The selected rooms vary in size, layout complexity, and object density, ranging from small office rooms to larger and more cluttered environments. This diversity allows for a comprehensive evaluation of the proposed method under different spatial conditions.

The data acquisition process was performed using a 360° panoramic camera mounted on a stick. The camera was moved along predefined paths to capture the entire room from multiple viewpoints. During this process, *IMU* and *GNSS/GPS* data were simultaneously recorded to support camera pose estimation. After data collection, the captured images were processed through the proposed pipeline, including 3D model reconstruction, refinement, and CCTV placement optimization. The optimized camera positions were then physically implemented in the real environment. Finally, the performance was evaluated using visibility coverage, blind spot reduction, and RMSE by comparing simulation results with real-world measurements.

### 5.4. Discussion

The experimental results demonstrate that the proposed indoor 3D reconstruction and CCTV placement optimization framework can generate accurate and practical camera placement recommendations. The low *RMSE* values observed across all test environments indicate that the reconstructed *digital twin* provides reliable spatial information for real-world deployment. A key factor contributing to this accuracy is the integration of *VSLAM-based pose estimation* with *IMU measurements,* which improves the stability of camera trajectory estimation during data acquisition. This improvement directly enhances the quality of the reconstructed 3D model.

In addition, the use of *3D Gaussian Splatting* enables the generation of a dense and continuous spatial representation, supporting precise visibility analysis during the optimization stage. Another important observation is that the optimization algorithm adaptively determines the number of allocated cameras for different indoor environments. This result indicates that the proposed method can automatically determine both the number of cameras and their placement positions without manual configuration. The recommended placements generated in the virtual environment can be effectively applied in real environments with minimal *positional error*, demonstrating the practicality of the proposed approach.

Conventional CCTV placement approaches, such as corner-based or uniformly distributed camera placement, can provide simple deployment strategies without requiring a reconstructed 3D environment. However, these heuristic methods generally do not consider complex indoor occlusions caused by walls, furniture, and other objects. In contrast, the proposed method can perform visibility analysis directly on the reconstructed *digital twin* using *ray-casting simulation*, enabling more accurate visibility evaluation and blind spot reduction in complex indoor environments.

The proposed framework requires an offline preparation stage consisting of the panoramic image acquisition, sensor synchronization, 3D reconstruction, and visibility simulation. In the experiments, the panoramic data acquisition process required approximately 5–10 min per room, depending on the room size and object density. The computational cost is mainly dominated by the *3D Gaussian Splatting* reconstruction stage, while the *greedy-based optimization* process is computationally lightweight once the digital twin model has been generated.

In this study, the reconstruction pipeline, including *VSLAM*-based pose estimation, *Gaussian*-based reconstruction, and model refinement, was performed offline using GPU acceleration. Although the reconstruction stage requires higher computational resources compared to conventional mapping methods, the generated *digital twin* provides dense spatial information that significantly improves the reliability of visibility analysis and CCTV placement optimization. Therefore, the computational overhead is considered acceptable for practical indoor surveillance planning applications.

However, this study is limited to relatively simple indoor environments with moderate *object density.* In more complex environments, such as large halls or heavily occluded spaces, additional cameras may be required to achieve sufficient coverage. Furthermore, the reconstruction accuracy depends on the quality of the captured panoramic images and sensor measurements. Future work will focus on improving the robustness of reconstruction and extending the optimization framework to larger and more complex indoor environments.

Although the proposed ray-casting visibility analysis considers camera viewing angles and indoor occlusions, several camera characteristics are simplified in the current implementation. Factors such as lens distortion, focal length variations, image quality degradation, and dynamic lighting conditions are not explicitly modelled. The current framework primarily evaluates geometric visibility coverage rather than detailed optical camera performance.

## 6. Conclusions

This paper presented a *digital twin-based framework* for indoor CCTV camera placement optimization using *360° panoramic image-based 3D reconstruction*. The proposed method integrates *panoramic data acquisition*, *VSLAM-based pose estimation* enhanced with *IMU measurements*, and *3D Gaussian Splatting* to generate a structured and accurate indoor model. Based on the reconstructed environment, a *ray-casting visibility analysis* combined with a *greedy optimization algorithm* was applied to determine optimal camera locations maximizing coverage while minimizing blind spots and overlap.

Experimental results across multiple indoor environments with varying layouts and complexities demonstrate that the proposed system achieves high visibility coverage and significant blind spot reduction. In addition, the *position accuracy evaluation* showed low *RMSE* values, indicating that the generated *digital twin* can provide reliable spatial coordinates for the deployment of real-world CCTV. The real implementation further confirmed that the optimized camera configuration can be applied directly in practical environments with consistent monitoring performance.

Overall, the proposed approach provides an efficient and practical solution for indoor surveillance planning by leveraging *digital twin technology* and *visibility-based optimization*. In future work, the proposed system will be extended to more complex and large-scale indoor environments, as well as integrated with *dynamic factors* such as human movements, lighting conditions, and real-time adaptive camera controls to further enhance surveillance performance.

In future work, semantic information will be integrated into the 3D model to enable context-aware CCTV placement. By incorporating object recognition and scene understanding, the system can prioritize important areas such as entrances, walkways, and critical assets, further improving surveillance effectiveness.

## Figures and Tables

**Table 1:** Input, processing, and output of each stage in proposed workflow.

Stage	Input	Processing	Output
Data Acquisition and Sensor Fusion (Section 3.2)	Indoor environment, IMU data, GNSS/GPS, camera parameters	360° image capture, sensor synchronization, trajectory recording	Panoramic images, sensor logs, spatial metadata
VSLAM-Based Indoor 3D Reconstruction (Section 3.3)	Panoramic images, IMU, GNSS/GPS	VSLAM-based pose estimation, Gaussian splatting reconstruction, global alignment	3D indoor model, point cloud, georeferenced environment
Strategic Model Refinement (Section 3.4)	Reconstructed 3D model	Point cloud upscaling, noise reduction, roof removal	Refined 3D model, cleaned point cloud
Greedy-based CCTV Placement Optimization (Section 3.5)	Refined 3D model, candidate camera positions	Ray-casting-based visibility simulation, blind spot detection, greedy optimization	Optimized camera layout, coverage metrics

**Table 2:** Details of indoor environments used in the panoramic dataset.

No.	Room ID	Room Type	Size (m)
a	D207	Laboratory Room	6.5 × 7.5
b	D306	Laboratory Room	6.5 × 7.5
c	D307	Laboratory Room	6.5 × 7.5
d	243	Laboratory Room	5 × 3 + 2 × 4
e	326	Pascasarjana Room	4.5 × 8
f	231	Aula Room	8.6 × 23
g	122	Laboratory Room	5.5 × 8.5
h	131	Library	6.75 × 14.5
i	233	Class Theory Room	5 × 8
j	111	Laboratory Room	7.15 × 7

**Table 3:** Visibility coverage and blind spot reduction results across different indoor environments.

Room	Coverage (%)	Blind Spot Reduction (%)
D207	92.4	85.1
D306	90.8	83.7
D307	93.1	86.5
243	91.5	84.2
326	94.2	87.8
231	96.8	91.2
122	92.7	85.9
131	95.1	89.4
233	93.8	87.3
111	94.5	88.6
Average	93.5	87.0

**Table 4:** Camera position accuracy evaluation using *RMSE* across 10 rooms.

Room	Recommended Cameras	RMSE (m)	Accuracy (%)
D207	2	0.18	96.5
D306	2	0.21	95.3
D307	2	0.17	96.9
243	2	0.19	95.8
326	2	0.16	97.2
231	8	0.23	94.5
122	3	0.20	95.5
131	6	0.18	96.4
233	2	0.22	94.9
111	4	0.24	94.1
Average	3.3	0.20	95.7

## Data Availability

Data are contained within the article.

## Abbreviations

CCTV	Closed-Circuit Television
GNSS	Global Navigation Satellite System
GPS	Global Positioning System
IMU	Inertial Measurement Unit
RMSE	Root Mean Squared Error
VSLAM	Visual Simultaneous Localization and Mapping
3DGS	3D Gaussian Splatting
